# Prophylactic administration of parecoxib sodium for postoperative delirium in hip surgery of the elderly: a prospective, randomized, placebo-controlled trial

**DOI:** 10.1590/1806-9282.20250548

**Published:** 2025-09-19

**Authors:** Qin Tan, Yingchuan Li, Quanhong Zhou

**Affiliations:** 1 Shanghai Jiaotong University Affiliated Sixth People's Hospital, Department of Critical Care Medicine – Shanghai, China.; 2 Tongji University Affiliated Tenth People's Hospital, Department of Critical Care Medicine – Shanghai, China.

**Keywords:** Postoperative delirium, Parecoxib sodium, Elderly, Orthopedic, Primary prevention

## Abstract

**OBJECTIVE:**

The aim of this study was to evaluate the effect of prophylactic administration of parecoxib sodium on postoperative delirium in elderly orthopedic patients.

**METHODS:**

In this prospective, single-center, randomized, double-blind, placebo-controlled trial, a total of 166 elderly orthopedic patients were included. Patients were randomly divided into placebo group (Group A) and parecoxib sodium group (Group B) with normal saline and parecoxib sodium 40 mg given, respectively, before operation. Delirium was evaluated by the Confusion Assessment Method-Chinese Reversion. The main outcome was the incidence of delirium within 48 h after operation. The secondary outcomes were the average delirium and pain scores. The follow-up outcome was measured by the Activities of Daily Living scale after 2 months and 6 months.

**RESULTS:**

Finally, 53 of 166 patients developed postoperative delirium (31.9%), of which the incidence of Group B was significantly lower than that of Group A (42.2 vs. 21.7%, p=0.015). The average delirium scores of Group B were lower than those of Group A (p<0.05). However, the average pain scores only differed at 8 pm in the second day after surgery. There was no significant difference in Activities of Daily Living scale scores between the two groups at either 2 months (67.12±19.82 vs. 65.19±19.53, p=0.609) or 6 months (68.36±18.56 vs. 66.63±19.44, p=0.927).

**CONCLUSION:**

Preoperative prophylactic administration of parecoxib sodium significantly reduced the incidence of postoperative delirium in elderly orthopedic patients but did not improve long-term functional recovery.

## INTRODUCTION

Postoperative delirium (POD) is common in elderly patients after surgery, mainly featuring as acute cognitive impairment, attention disorder, and reversible thinking disturbance^1^. The incidence of POD in hip arthroplasty has been reported to range from 21 to 69%, which increases morbidity and mortality, prolongs length of hospital stay, and lays financial burden on society and family^2,3^.

Although it has been studied for many years, the specific pathophysiological mechanism of POD is still unknown. At present, the systemic inflammatory response and pain-related stress are believed to be the main causes of postoperative mental disorder^4,5^. Under various stimulations, leukocytes proliferate and adhere to the endothelial cells of the blood–brain barrier (BBB), releasing reactive oxygen species and proteases^6^. Pro-inflammatory factors cross the BBB and enter the brain, leading to an inflammatory cascade effect^7^.

Parecoxib sodium, one kind of non-steroidal anti-inflammatory drugs (NSAIDs), is the first selective inhibitor of cyclooxygenase-2 (COX-2) for parenteral use. Its selectivity to COX-2 is about 28,000 times that of COX-1. Studies have shown that the administration of parecoxib sodium (40 mg at the end of surgery and twice daily for 3 days) could significantly reduce the incidence of delirium (from 11.0 to 6.2%) after hip or knee replacement surgery and a single dose of parecoxib administration could also relieve postoperative pain, reduce the use of morphine, and alleviate confusion^8^. However, few studies have reported the short-term and long-term effects of a single dose of preoperative parecoxib sodium on POD in elderly patients.

We hypothesized that the prophylactic administration of parecoxib sodium could decrease the incidence of POD and improve long-term prognosis.

## METHODS

This was a prospective, randomized, double-blinded, placebo-controlled, single-center clinical trial. The study was registered at the Chinese Clinical Trials Registry (ChiCTR1800019988) and was performed in the Shanghai Sixth People's Hospital. The study protocol was approved by the Ethics Committee of the Shanghai Sixth People's Hospital (2019–029). Written informed consent was obtained from every patient. This trial was conducted in accordance with the Declaration of Helsinki.

### Patients

Patients were screened the day before surgery using their medical records, and at the same time, their basic information and baseline characteristics were collected, including hospitalization number, name, gender, age, contact information, education level, height, weight, heart rate, blood pressure, and comorbid diseases.

After preliminary screening, a researcher who was blinded to the groups administered the Abbreviated Mental Test Score (AMTS) to assess the patients’ cognitive function. The AMTS consists of seven items, with a total score of 10. A score less than 5 indicates severe cognitive impairment. The AMTS has a good consistency with commonly used tools, such as the Mini-Mental State Examination (MMSE) and Montreal Cognitive Assessment (MoCA), in the diagnosis of objective cognitive impairment. Moreover, compared with these tools, AMTS is much faster and more convenient in practice and has lower requirements on the patient's hearing, vision, and education level^9^.

The inclusion criteria were elderly patients (age ≥70 years), weighing over 50 kg, who underwent any type of elective hip surgery.

Patients were excluded if they met any of the following criteria:

1. presence of severe cognitive impairment before operation;
2. presence of severe coronary artery disease (defined as left ventricular ejection fraction ny t has lower requirements on the impairment. Moreover, compared w, or a previous history of coronary artery bypass grafting;
3. those with serious hearing or visual impairment, unable to communicate;
4. presence of severe liver dysfunction, with child Pugh score ≥10;
5. presence of malignant tumors;
6. presence of active gastrointestinal bleeding or inflammatory bowel disease;
7. those allergic to NSAIDs or sulfonamides;
8. those who refused to sign consent.

### Simple size and randomization

Based on the previous studies, we hypothesized that 40% of the patients in the placebo group (Group A) had the occurrence of POD compared to 20% of the patients in the parecoxib sodium group (Group B)^10^. According to the sample size formula for two independent groups, we set the alpha level at 5% and power at 80% and expected the lost to follow-up rate at 10%. After calculation, each group required a total of 82 patients to be able to detect a difference.

Patients who met the test conditions and provided informed consent were randomly assigned to placebo group (Group A) or parecoxib sodium group (Group B). Randomization was performed using a block size of 4 with a 1:1 allocation ratio. The randomized block design was used to control the possible differences between the two groups so as to ensure the symmetry and comparability of the sample size of the two groups. Double-blinding was ensured by identical packaging and placebo preparation by an independent pharmacist. Specific grouping was performed by the clinical pharmacists of the hospital, and the relevant researchers and patients were blinded to the groups.

### Study drug administration

After randomization, the patients received placebo or parecoxib sodium: Group A received placebo (5 mL 0.9% sodium chloride injection) intravenously after general anesthesia and before skin incision, while Group B received 40 mg parecoxib sodium (dissolved in 5 mL 0.9% sodium chloride injection) intravenously. The preparation process of the drug was carried out by the clinical pharmacists, and the researchers could not distinguish the difference when they administered the drug.

### Outcome assessment

#### Primary outcome

The enrolled patients were assessed at 8 am, 12 pm, and 4 pm in the first two days after surgery, totaling six assessments. The diagnosis of POD was based on the Confusion Assessment Method-Chinese Reversion (CAM-CR), which has been validated in perioperative Chinese populations^11,12^. The CAM-CR assessed four basic characteristics of delirium, such as acute disturbance of consciousness, attention, cognition, and perception, using a total of 11 specific items. Each item is scored from 0 to 4 according to the severity and changes in the symptoms of patients. The total score is then used to determine the presence of delirium, with a score greater than 22 indicating a diagnosis of POD.

#### Secondary outcome

Postoperative pain was assessed using the Faces Pain Scale-Revised (FPS-R) at fixed time points: 8 am, 12 pm, and 4 pm on the first two postoperative days. The FPS-R was used for patients who could not speak and had a low education level. In a small-sample study comparing pain scales among individuals with cognitive impairment, 54% of the participants preferred the FPS-R for pain assessment^13^.

#### Follow-up

The Activities of Daily Living (ADL) scale was used to evaluate the prognosis^14^. The ADL scale evaluates 10 items of basic personal activities, scoring from 0 (complete dependence) to 10 (complete independence), with a total score of 100 points. A score of 100 is regarded as completely independent, 61 or more as mild functional impairment, 41–60 as moderate functional impairment, and 40 or less as severe functional impairment. Patients were followed up by telephone 60 days after discharge, with the follow-up made on issues related to ADL.

### Statistical analysis

Normally distributed continuous variables were presented as mean±standard deviation and were compared between the study groups using independent t-test. Continuous variables that were not normally distributed were presented as median with interquartile range and were statistically analyzed using the Mann-Whitney test. Categorical variables, presented in counts or as percentages, were analyzed using the chi-square or Fisher's exact test when appropriate. The data were analyzed using SPSS software, Version 24.0 (IBM Corp, Armonk, NY, USA). A two-sided p<0.05 was considered to be statistically significant.

## RESULTS

**Figure 1:**
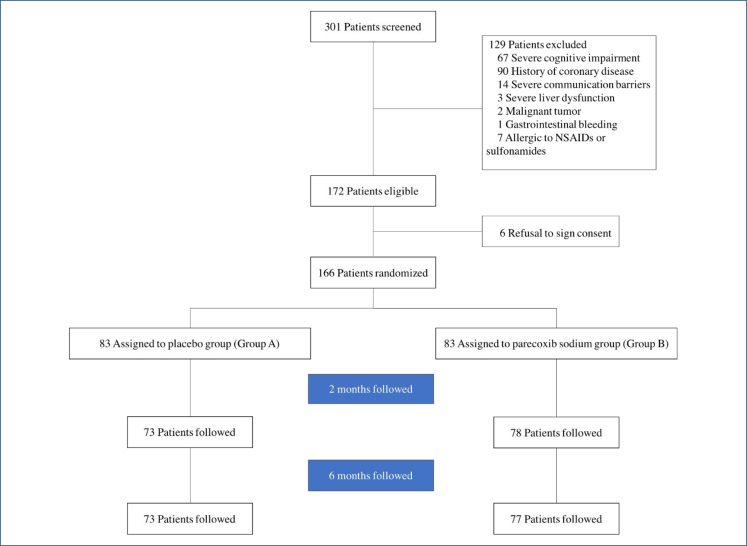
Flowchart of the study. NSAIDs: non-steroidal anti-inflammatory drugs.

From June 6, 2019, to March 31, 2020, a total of 301 patients were screened. Of these, 135 were not enrolled: 129 patients met the exclusion criteria and 6 declined to provide consent (Figure 1). The remaining 166 patients were randomized into two groups, with 83 assigned to the placebo group (Group A) and 83 to the parecoxib sodium group (Group B). At 2-month follow-up, 73 (87.9%) patients in Group A and 78 (94.0%) patients in Group B completed the data collection procedure, while at 6-month follow-up, 73 (87.9%) patients in Group A and 77 (92.8%) patients in Group B completed the data collection procedure.

The baseline characteristics are presented in Table 1. Among the enrolled patients, 87.2% were admitted to the hospital for femur neck fracture.

**Table 1:** Baseline characteristics of the patients.

		Placebo (n=83)	Parecoxib sodium (n=83)	p-value
Male, n (%)		31 (37.3)	36 (43.4)	0.429
Age, mean±SD		79.94±7.57	80.18±6.82	0.830
Height, mean±SD (cm)		161.22±7.11	161.65±7.49	0.703
Weight, mean±SD (kg)		59.63±9.62	58.74±9.25	0.541
Heart rate, mean±SD (bpm)		78.45±11.28	76.98±12.17	0.421
SBP, mean±SD (mmHg)		131.89±18.36	137.90±21.26	0.053
DBP, mean±SD (mmHg)		70.83±11.79	71.88±13.61	0.597
AMTS, mean±SD		8.00±1.59	7.82±1.52	0.454
Comorbidities, n (%)				
	Hypertension	42 (50.6)	42 (50.6)	1.000
	Diabetes	17 (20.5)	17 (20.5)	1.000
	Hemiplegia	0 (0.0)	0 (0.0)	
	COPD	17 (20.5)	12 (14.5)	0.307
	Coagulopathy	0 (0.0)	0 (0.0)	
	Arrhythmia	19 (22.9)	20 (24.1)	0.855
Surgical type, n (%)				
	Femoral neck fracture repair	40 (48.2)	42 (50.6)	0.73
	Hip arthroplasty	4 (4.8)	3 (3.6)	0.78
	Acetabular fracture fixation	2 (2.4)	1 (1.2)	0.62
Education level, n (%)				
	No formal education	36 (43.4)	29 (34.9)	0.266
	Completed primary school	20 (24.1)	21 (25.3)	0.857
	Completed high school	22 (26.5)	21 (25.3)	0.859
	Completed graduation	5 (6.0)	12 (14.5)	0.073

SBP: systolic blood pressure; DBP: diastolic blood pressure; AMTS: Abbreviated Mental Test Score; SD: standard deviation; COPD: chronic obstructive pulmonary disease.

### Primary outcome

Among the 166 patients enrolled, after six POD assessments, a total of 53 patients had a CAM-CR score of more than 22 points, which indicated the presence of POD. Therefore, the overall incidence of delirium in this study was 31.9%, which is similar to the data reported in the previous literature^10,15^.

A total of 35 patients from Group A and 18 from Group B presented with POD. There was a significant different in the incidence between the two groups (p=0.015) (Table 2).

**Table 2:** Primary and secondary outcomes.

		Placebo (n=83)	Parecoxib sodium (n=83)	p-value
Primary outcome				
Delirium incidence, n (%)		35 (42.2)	18 (21.7)	0.015*
CAM-CR, mean±SD				
Day 1	8 am	18.87±4.67	16.95±3.73	0.003*
	12 pm	18.75±4.31	17.11±3.48	0.020*
	4 pm	18.65±4.52	16.83±3.61	0.003*
Day 2	8 am	19.05±4.29	17.11±3.57	0.002*
	12 pm	18.99±3.98	16.58±3.10	0.001*
	4 pm	18.72±4.00	16.06±2.72	0.001*
**Secondary outcome**				
FPS-R, mean±SD				
Day 1	8 am	2.23±1.14	2.27±0.94	0.824
	12 pm	2.25±1.03	2.13±1.00	0.446
	4 pm	2.27±1.02	2.22±0.99	0.758
Day 2	8 am	2.43±1.01	2.13±0.93	0.048*
	12 pm	2.25±0.97	2.12±0.93	0.371
	4 pm	1.98±0.95	1.95±0.85	0.864

CAM-CR: Confusion Assessment Method-Chinese Reversion; FPS-R: Faces Pain Scale-Revised; SD: standard deviation.

* p<0.05.

### Secondary outcome

All patients received femoral nerve block analgesia before operation, and there was no significant difference in the average score of postoperative pain at three time points on the first day. Only in the morning of the second day after operation, the average score of Group A was 2.43±1.01, while that of Group B was 2.13±0.93 (p=0.048), with statistical difference (Table 2).

### Follow-up

Among all cases that completed follow-up, there was no significant difference in ADL scale scores between the two groups at either 2 months (67.12±19.82 vs. 65.19±19.53, p=0.609) or 6 months (68.36±18.56 vs. 66.63±19.44, p=0.927). Patients with POD demonstrated significantly lower ADL scale scores compared to non-POD patients (65.64±21.73 vs. 67.14±14.47, p=0.015), suggesting a clinically meaningful impairment in functional independence.

### Logistic regression analysis

The results demonstrated that the drug group remained independently associated with POD after adjusting for these covariates: compared with placebo group, the parecoxib sodium group was associated with a lower risk of incident POD (adjusted OR 0.25, 95%CI 0.11, 0.58; p=0.001). Detailed results are presented in Table 3.

**Table 3:** Multivariable logistic regression analysis of potential predictors of postoperative delirium.

Primary outcome (POD)		Odds ratio (95%CI)	p-value
Parecoxib sodium group		0.25 (0.11–0.58)	0.001
Male		2.29 (0.68–7.69)	0.179
Age		1.07 (1.00–1.14)	0.047
Height		1.03 (0.94–1.12)	0.565
Weight		0.98 (0.93–1.03)	0.406
Heart rate		1.03 (0.99–1.07)	0.100
SBP		0.99 (0.97–1.02)	0.579
DBP		0.99 (0.95–1.03)	0.649
AMTS		0.78 (0.57–1.08)	0.133
Comorbidities			
	Hypertension	1.06 (0.48–2.35)	0.888
	Diabetes	3.75 (1.36–10.38)	0.011
	COPD	2.32 (0.67–7.99)	0.184
	Arrhythmia	2.44 (0.95–6.30)	0.065
Education level			
	No formal education	1.00 (reference)	-
	Completed primary school	1.95 (0.68–5.58)	0.215
	Completed high school	1.36 (0.47–3.99)	0.570
	Completed graduation	0.72 (0.16–3.30)	0.673

CI: confidence interval; POD: postoperative delirium; SBP: systolic blood pressure; DBP: diastolic blood pressure; AMTS: Abbreviated Mental Test Score; COPD: chronic obstructive pulmonary disease.

## DISCUSSION

This prospective, randomized, double-blind, placebo-controlled trial aimed to evaluate the effect of prophylactic use of parecoxib sodium on POD and prognosis in elderly orthopedic patients. The results demonstrated that (1) preoperative administration of parecoxib sodium could significantly reduce the incidence of POD in elderly orthopedic patients, and it was safe without obvious adverse reactions; (2) the occurrence of POD would affect the long-term quality of life of patients; and (3) a single dose of parecoxib sodium could not directly affect the prognosis of patients. Therefore, our research showed that prophylactic administration of parecoxib sodium might be used to prevent POD in elderly orthopedic patients.

Current evidence underscores that neuroinflammation driven by central nervous system (CNS) immune activation is a pivotal mechanism underlying POD. Preclinical studies reveal that surgical trauma disrupts the BBB, facilitating inflammatory cytokine infiltration and microglial activation^16^. Animal experiments have shown that inflammatory signals crossing the damaged BBB activate microglia, triggering immune responses such as phagocytosis, antigen presentation, rapid proliferation, and secretory mediators. And finally, these processes would weaken the tight junction of astrocytes, impairing neural function^17^. Notably, aged animals exhibit elevated baseline neuroinflammatory cytokines and hyperactive microglia, rendering them susceptible to POD^18^.

The interplay between COX-2-mediated inflammation and neuronal vulnerability underpins POD pathogenesis. Surgical stress induces phospholipase A2 activation, liberating arachidonic acid for prostaglandin synthesis. Prostaglandin E2 (PGE2) amplifies nociceptive signaling via EP3 receptors, exacerbating postoperative pain, while simultaneously activates NLRP3 inflammasomes to drive neuroinflammation^19,20^. Parecoxib sodium, a selective COX-2 inhibitor, demonstrates dual neuroprotective effects:

Anti-inflammatory action: By inhibiting PGE2 synthesis, it suppresses microglial overactivation and reduces pro-inflammatory cytokine release (e.g., interleukin [IL]-1β and tumor necrosis factor [TNF]-α)^21^.

Direct neuronal protection: In vitro studies show that parecoxib upregulates anti-apoptotic Bcl-2 family proteins, stabilizes mitochondrial membrane permeability, and attenuates hypoxia-induced neuronal death. In vivo experiments further confirm its ability to reduce astrocyte activation and neurotoxic factor release, thereby preserving cognitive function in rodent models^22^.

In this study, parecoxib sodium was used in elderly orthopedic patients before operation, and some meaningful results were obtained. The data showed that the use of parecoxib sodium significantly reduced the incidence of POD in elderly orthopedic patients (42.2 vs. 21.7%, p=0.015). While parecoxib's analgesic properties may transiently improve early postoperative pain scores (2.27±1.02 vs. 2.13±0.93, p=0.048), this effect does not equate to direct POD prevention. The study's follow-up results showed that a single dose of parecoxib sodium had no significant effect on the prognosis of patients, which may indicate that to improve the prognosis of patients, it is necessary to further extend the medication time of parecoxib sodium.

There are several limitations in this study. First of all, this study was a single-center clinical study. The data of this study were only collected from the orthopedic ward of the same hospital, and therefore could not represent the whole population of patients with delirium. Second, this study did not collect the relevant inflammatory biomarkers (e.g., IL-6 and TNF-α), which limits the mechanistic insight into how parecoxib sodium modulates neuroinflammation. Future trials should incorporate serial measurements of peripheral and central inflammatory markers to elucidate the pharmacodynamic effects of COX-2 inhibition on POD. Third, our 48-h follow-up period may underestimate late-onset POD, which typically peaks between postoperative days 3 and 5, and missing follow-up data might affect the final results. Finally, our 6-month follow-up period may be insufficient to fully capture delayed neurocognitive decline, particularly in vulnerable elderly populations. Prolonged observation (e.g., 12ammatory markers to elucidate the pharmacodynamic effects of COX-2

## CONCLUSION

Preoperative prophylactic administration of parecoxib sodium significantly reduced the incidence of POD in elderly orthopedic patients. However, this single-dose regimen did not demonstrate long-term benefits on functional recovery.

## Data Availability

The datasets generated and/or analyzed during the current study are available from the corresponding author upon reasonable request.
